# Low Oxygen Availability Increases Itaconate Production by *Ustilago maydis*

**DOI:** 10.1002/bit.70035

**Published:** 2025-08-02

**Authors:** Marianne Volkmar, Wolfgang Laudensack, Felix Bartzack, Niklas Erdmann, Sonja Schönrock, Emely Fuderer, Dirk Holtmann, Lars M. Blank, Roland Ulber

**Affiliations:** ^1^ Institute of Bioprocess Engineering University of Kaiserslautern‑Landau Kaiserslautern Germany; ^2^ Karlsruhe Institute of Technology Institute of Process Engineering in Life Sciences Karlsruhe Germany; ^3^ ABBt – Aachen Biology and Biotechnology, iAMB ‐ Institute of Applied Microbiology RWTH Aachen University Aachen Germany

**Keywords:** itaconic acid, O_2_ limitation, scale up, *Ustilago maydis*

## Abstract

Itaconic acid is a monomer for high performance polymers. While produced in industry with the filamentous fungi *Aspergillus terreus*, the production with the smut fungus *Ustilago maydis* was proposed recently. The strict aerobic process suffers from high power input via gassing and stirring. Here, we investigated in detail possible scenarios for the reduction of energy use during cultivation. In contrast to fermentations with other organic acids, in which even small oxygen concentrations in the medium hinders production, low oxygen availability correlated with increased itaconic acid titer and yield. This is somewhat surprising, as not only the sensitivity of itaconic acid producing *U. maydis* to oxygen deprivation was previously reported, but also the lower degree of reduction compared to glucose is not directly arguing for improved yield. Scale ups from 0.4 to 30 L using different criteria confirmed the positive impact of low availability of oxygen on itaconic acid production, suggesting a shift in metabolic pathways under restricted oxygen conditions. Oxygen limitation, encountered more likely in industrial fermenters, can be effectively used as a process control strategy to enhance itaconic acid production in *U. maydis*, offering a new approach for improving the efficiency of industrial‐scale biotechnological production.

## 1 Introduction

**Figure 1:**
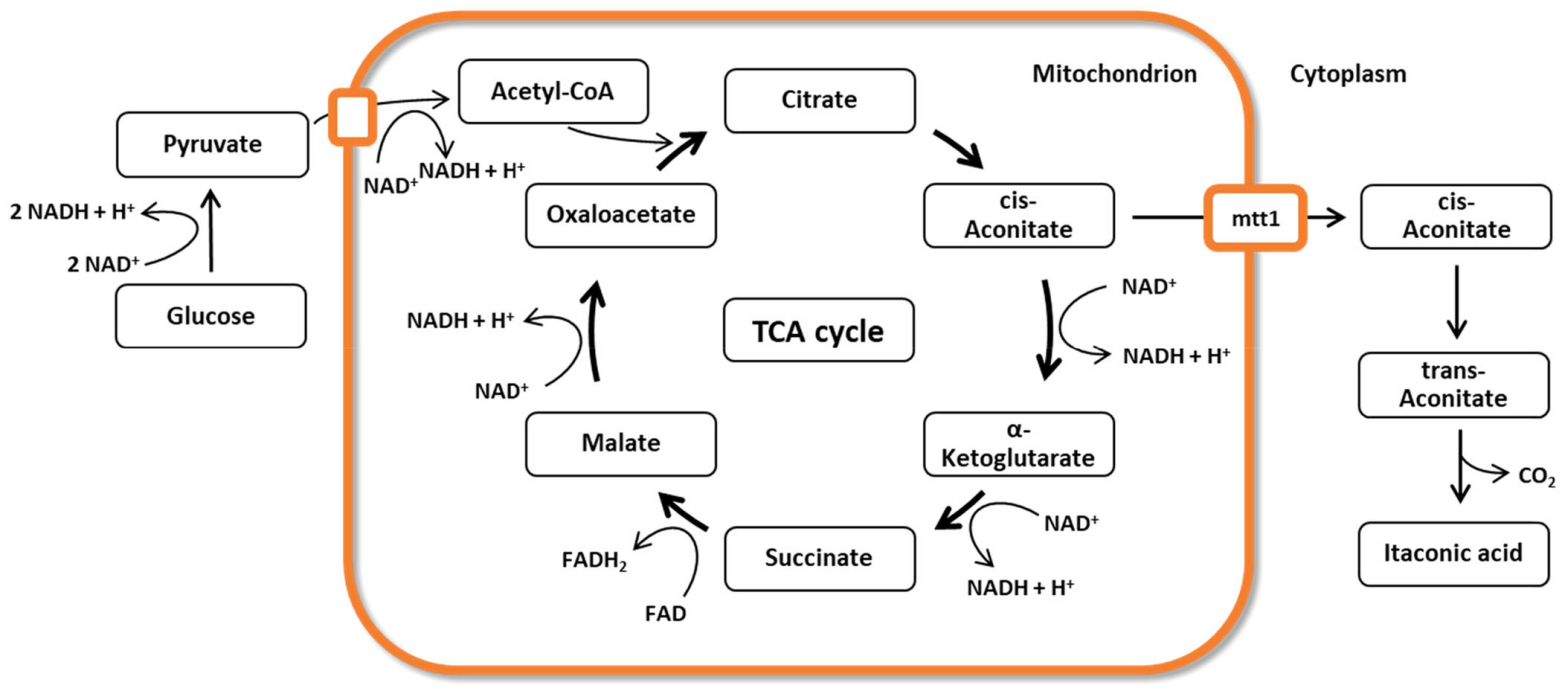
Simplified scheme of itaconic acid production in *U. maydis*, based on (Geiser et al. 2016b).

Itaconic acid is an unsaturated dicarboxylic acid. It is primarily used as a co‐monomer for acrylic polymers and rubbers (Teleky and Vodnar 2021), but also for the production of binders, thickeners, resins and coatings (Willke and Vorlop 2001). Due to its structural similarity to acrylic acid and methacrylic acid, it is suitable to replace these petrochemical‐based components (Kuenz and Krull 2018). This makes it a promising platform chemical for a sustainable industry. So far, compared to the global demand of 6.2 · 10^6^ t for acrylic acid, the market volume of itaconic acid is rather small with 41,400 t per year (Kuenz and Krull 2018). However, the current market value of 117.8 mio US$ (2024) is projected to grow by 7.8% until 2029 (Market Data Forecast 2023). The commercial production of itaconic acid happens exclusively via biotechnological routes (Willke and Vorlop 2001; Kuenz and Krull 2018). The established organism for this purpose is the filamentous fungus *Aspergillus terreus*, enabling itaconic acid titers of up to 160 g ∙ L^−1^, production rates of 1.53 g ∙ L^−^^1^ ∙ h^−1^, and yields of 0.63 g_ITA_ ∙ g_GLU_^−1^, which is close to the maximum theoretical yield of 0.72 g_ITA_ ∙ g_GLU_^−1^ (Becker et al. 2020a; Krull et al. 2017). However, due to its filamentous growth and the resulting shear sensitivity (Tehrani et al. 2019; Klement et al. 2012), the cultivation of *A. terreus* is challenging. In the course of the development towards a sustainable industry, the inclusion of residual flows such as agricultural residues is of particular interest. The sensibility of *A. terreus* towards impurities in the medium prevents the use of agricultural residues as renewable feedstock (Bafana and Pandey 2018). This is why the smut fungus *Ustilago maydis* is currently considered to be an alternative itaconic acid producer (Becker et al. 2020a). Compared to *A. terreus*, *U. maydis* has some advantages as a production organism. Firstly, it is able to grow in single cells instead of filamentously. Furthermore, it has also been shown that it can grow on sustainable substrates (Weiermüller et al. 2021; Volkmar et al. 2023). As a model organism for plant pathogenesis (Kämper et al. 2006), it displays a dimorphic life cycle (Bölker 2001). The haploid spores are non‐pathogenic and grow yeast‐like, making it possible to cultivate them in media (Börner 2009). The conjugation of haploid spores leads to the infectious, filamentous dicaryot (Kahmann and Kämper 2004). Besides itaconic acid, it also produces 2‐hydroxyparaconic acid, glycolipids, iron‐chelating siderophores, indol pigments, and other organic acids such as ustilagic or malic acid (Becker et al. 2020a; Becker et al. 2020b; Bölker 2001; Bölker et al. 2008; Zambanini et al. 2017; Budde and Leong 1989; Zuther et al. 2008). The organism *U. maydis* MB215 ΔCyp3 P_etef_Ria1 used in this study was genetically modified to suppress side product formation. The deletion of the cyp3 gene prevents the degradation of itaconic acid to 2‐hydroxyparaconic acid, while the promotor exchange leads to an overexpression of the gene cluster for itaconic acid production (Tehrani et al. 2019). Figure 1 shows a simplified metabolism of *U. maydis* for the production of itaconic acid. Cis‐aconitate from the tricarboxylic acid (TCA) cycle is exported from the mitochondrion via the mtt1 transporter, converted into itaconic acid in the cytoplasm and exported to the extracellular space via the Itp1 transporter (Zambanini et al. 2017; Geiser et al. 2016b). In contrast to *A. terreus*, where itaconic acid is produced directly from cis‐aconitate, itaconic acid production in *U. maydis* takes place in two steps via the intermediate trans‐aconitate (Geiser et al. 2016b).

To consider itaconic acid production via *U. maydis* as economically worthwhile process, a scale up of the fermentation is essential to increase the production volume. However, the scale impacts biological factors such as the number of cell divisions during inoculum preparation and the sensitivity to contaminations. Chemical and physical factors are affected by the scale as well, for example the means of pH control, the redox potential, temperature control, aeration, and agitation (Junker 2004). Common scale up approaches include constancy of volumetric mass transfer coefficient (k_L_a), power input per volume of medium, mixing time, and impeller tip speed (Mello et al. 2024; Junker 2004). One scale up with *U. maydis* has been performed based on constant dissolved oxygen tension (DO) (Helm et al. 2024). However, applied DO levels for fermentations with *U. maydis* fluctuate widely in the literature between 30% and 80% (Helm et al. 2024; Becker et al. 2020b; Geiser et al. 2016a; Ullmann et al. 2021), indicating a lack of clarity of the exact influence of DO. Therefore, this paper aims to investigate scale up criteria for the production of itaconic acid using *U. maydis* and explores the influence of oxygen availability during fermentation.

## 2 Materials and Methods

### 2.1 Cultivation

The genetically modified strain *Ustilago maydis* MB215 ΔCyp3 P_etef_Ria1 was designed in the working group Blank (RWTH Aachen, Germany) (Tehrani et al. 2019). It was stored as cryo culture at −80°C with 40% glycerol. Pre‐cultures were grown in shake flasks with baffles at 30°C and 120 rpm for 48 h in YEPG medium containing 10 g ∙ L^−1^ yeast extract, 20 g ∙ L^−1^ casein peptone, 20 g ∙ L^−1^ glucose, and 167 mg∙L^−1^ streptomycin sulfate. For screening experiments, pre‐cultures were grown in 500 mL shake flasks while the pre‐cultures for scale up experiments were cultivated in 1 L shake flasks. In both approaches, the flasks were filled to 30% with YEPG medium. Main cultures were inoculated to OD_600_ 0.5 and grown in modified Tabuchi medium. It contains 19.5 g ∙ L^−1^ MES buffer, 50 g ∙ L^−1^ glucose, 0.8 g ∙ L^−1^ ammonium chloride, 0.5 g ∙ L^−1^ monopotassium phosphate, 0.2 g ∙ L^−1^ magnesium sulfate heptahydrate, 0.1 g ∙ L^−1^ iron sulfate heptahydrate, vitamins, and trace elements at pH 6.5 (Geiser et al. 2014). Screening experiments were conducted in the 0.5 L reactor system Biostat Q+ from Sartorius (Göttingen, Germany) with a working volume of 0.4 L. The scale up was carried out in the 42 L reactor system Biostat C+ (Sartorius, Germany) with a working volume of 30 L. A dimensioned sketch of the reactors including the reactor internals is depicted in Figure S1. Both reactors have six‐bladed Rushton impellers and a ring sparger. The Biostat C+ reactor is equipped with a VISIFERM DO 120 probe (Hamilton, Bonaduz, Switzerland) and a BioPAT Xgas sensor (Sartorius, Germany), measuring CO_2_ via infrared and O_2_ via lambda probe in the off‐gas. Antifoam was added manually (Biostat Q + ) or automatically (Biostat C + ) as needed.

### 2.2 Analytical Methods

The volumetric oxygen transfer coefficient k_L_a was determined via degassing method in distilled water and modified Tabuchi medium at 30°C. Oxygen concentration was determined using an OxyFerm FDA 120 probe (Hamilton, Bonaduz, Switzerland) in both reactor systems. k_L_a was calculated by integrating the general oxygen equation of an aerobic fermentation as shown in equation (1).

(1) $$\ln\left(\frac{{c_{0}}^{*}}{{c_{0}}^{*}-c_{0}}\right)=k_{L}a∙t,$$

With c_0_* = saturation concentration of the liquid phase and c_0_ = O_2_ concentration in the liquid phase.

The k_L_a corresponds to the slope of the graphical representation of equation (1) over time.

Biomass growth was monitored by off‐line measuring the optical density at 600 nm (OD_600_) using a CO8000 Cell Density Meter (Biochrom Ltd. Cambridge, England; 1 OD_600_ = 0.204 g_CDW_·L^−1^) or a LAMBDA Bio + spectrophotometer (Perkin Elmer Corporation, Waltham, USA; 1 OD_600_ = 0.139 g_CDW_·L^−1^), with OD_600_ values converted to cell dry weight (CDW) based on the respective device‐specific correlation. Substrate and product concentration were analyzed via a HPLC system (ESA Inc. 542 autosampler (Chelmsford, Massachusetts, USA), Azura pump P 6.1 L (Knauer GmbH, Berlin, Germany)) with a refractive index detector (RI 101 Shodex, Showa Denke KK, Tokyo, Japan). The Bio‐Rad Aminex HPX‐87H column (300 × 7.8 mm, Hercules, California, USA) had a temperature of 80°C. 2.5 mM H_2_SO_4_ with a flow rate of 0.6 mL∙min^−1^ was used as the mobile phase. As the buffer substance of the medium MES is chemically stable and is not metabolized during the cultivation, its concentration was used as internal standard to account for any changes in concentration due to evaporation or dilution by correctives. The correction factor was used for the concentration of glucose and itaconic acid as well as for the CDW.

### 2.3 Calculation

The amount of carbon bound in the biomass and the volume flow and amount of CO_2_ were determined to calculate the carbon balance. To calculate the former, the established relation between optical density and cell dry weight was calculated according to Equation (2).

(2)

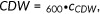

with an obtained proportionality factor c_CDW_ of 0.20395 ± 0.01717 g ∙ L^−1^. The amount of carbon bound in the biomass was adopted from Klement et al. reporting a share of 0.579 g ∙ g^−1^ (Klement et al. 2012), which leads to Equation (3).

(3) $$m_{C,\mathrm{CDW}}\left(t_{E}\right)=\left({\mathrm{OD}}_{600}\left(t_{E}\right)-{\mathrm{OD}}_{600}\left(t_{0}\right)\right)∙c_{\mathrm{CDW}}∙V_{R}∙0.579g∙g^{-1}$$

*t_E_* = time of measurement, *t_0_* = start time, *V_R_* = reaction volume

The volume flow ${\dot{V}}_{\text{C}{\text{O}}_{2}}(\text{t})$ was calculated according to equation (4), considering an exhaust gas flow of 5 ∙ 10^‐4 ^m³∙s^−1^.

(4) $${\dot{\text{V}}}_{\text{C}{\text{O}}_{2}}(\text{t})=5∙10^{-4}{\text{m}}^{3}∙{\text{s}}^{-1}\;∙\;{\text{X}}_{\text{C}{\text{O}}_{2}}^{\left(\text{v}\right)}(\text{t}),$$

$X_{\text{C}{\text{O}}_{2}}^{\left(\text{v}\right)}(t)$ = measured volume fraction of CO_2_ in the exhaust gas.

To calculate the flow of CO_2_ in the exhaust gas, Equation (5). was applied, assuming an exhaust gas temperature of 20°C due to the exhaust gas cooler.

(5) $${\dot{\text{n}}}_{\text{C}{\text{O}}_{2}}(\text{t})=\frac{\text{p}\;∙{\dot{\text{V}}}_{\text{C}{\text{O}}_{2}}}{\text{R}∙\text{T}}=\frac{10^{5}\text{Pa}∙{\dot{\text{V}}}_{\text{C}{\text{O}}_{2}}(\text{t})}{\text{R}∙\left(20+273.15\right)\text{K}},$$

${\dot{n}}_{\text{C}{\text{O}}_{2}}(\text{t})$ = flow of amount of substance, *p* = pressure in the head space of the reactor, *R* = universal gas constant.

Integration of Equation (5) lead to Equation (6) yielding the amount of CO_2_.

(6) $${\text{n}}_{{\text{CO}}_{2}}{(\text{t}}_{\text{E}})=\sum_{{\text{t}}_{\text{i}\;}=\;{\text{t}}_{0}}^{{\text{t}}_{\text{E}}}{\dot{\text{n}}}_{\text{C}{\text{O}}_{2}}\left({\text{t}}_{\text{i}}\right)∙\Delta t.$$

## 3 Results and Discussion

### 3.1 Investigating Aeration of *U. maydis* for High Itaconic Acid Production

Screening experiments were conducted in 0.4 L bioreactors to find suitable process parameters for the cultivation of *Ustilago maydis*. The aim of the screening was to increase the concentration and yield of itaconic acid. The varied parameters were gassing rate and impeller speed. The impeller speed was varied in five steps from 300 to 1200 rpm with a flow of pressured air of 1 and 2.5 vvm. The tested range was analyzed according to Doran concerning impeller flooding and complete gas dispersion. The points of impeller flooding are 140 rpm for 1 vvm and 190 rpm for 2.5 vvm. Complete gas dispersion occurs from 330 rpm and 200 rpm, respectively. (Doran 2013). Therefore, no impeller flooding took place in the observed range. Both impeller speed and gassing rate influence the oxygen availability in the reactor, which is likely to impact the itaconic acid production due to the aerobic metabolism of *U. maydis* (Kuenz and Krull 2018). Table 1 summarizes amount of biomass, yield of itaconic acid per consumed glucose, space time yield after 40 h (time point chosen to make sure that there is no substrate depletion, which started for some experiments after 48 h), final concentration of itaconic acid after 108 h, and k_L_a.

**Table 1:** Summary of the screening experiments in 0.4 L scale.

Gas flow	Impeller speed	max CDW		product yield	STY (40 h)		final ITA conc.	k_L_a
	/rpm	/g_CDW_·L^−1^		/g_ITA_ ∙ g_GLU_^−1^	/g ∙ L^−1^ ∙ h^−1^		/g ∙ L^−1^	/h^−1^
1 vvm	300	5.75		0.48	0.17		26.3	40.4
	600	9.40		0.35	0.24		17.4	77.0
	800	9.14		0.32	0.23		16.5	96.6
	1000	9.61		0.32	0.22		16.4	123.3
	1200	9.22		0.31	0.21		16.2	142.7
2.5 vvm	300	6.55		0.47	0.21		22.9	58.2
	600	9.34		0.35	0.19		16.9	98.2
	900	9.16		0.35	0.27		19.6	162.5
	1000	9.10		0.34	0.21		17.1	183.3
	1200	8.06		0.33	0.21		19	236.6
1 vvm	53–929 (30% DO)	8.62		0.32	0.21		16.9	n.d.

*Note:* Experiments are grouped by gassing rate, with separate sections for constant impeller speed and for constant DO. Shown are biomass growth (CDW), yield, space time yield (40 h), and concentration of itaconic acid produced. n = 1. n.d.: not determined.

The course of the cultivation at 1 vvm and 300 rpm, which resulted in the highest final itaconic acid concentration, is shown as an example in Figure 3A. Cell growth started for all experiments within the first 12 h. For the experiment depicted in Figure 3A, biomass increased until around 99 h, while for most other parameter combinations from Table 1 the maximum CDW was reached at around 24 h. The substrate concentration decreased linearly until 114 h. For the experiment with 2.5 vvm and 300 rpm, glucose was depleted after 72 h, while for the rest of the tested combinations substrate depletion occurred after 48 h. *U. maydis* is a potential candidate to produce itaconic acid using media based on renewable resources, which often do not exceed carbon concentrations of 50 g ∙ L^−1^. This is why the focus in this study was on lower initial glucose concentrations. Itaconic acid production started after around 12 h. For most experiments, the itaconic acid concentration further increases after the depletion of glucose, which is accompanied with a decrease in CDW. This leads to the assumption that the production of itaconic acid is based on the degradation of the biomass. The literature reports the ability of *U. maydis* to metabolize nutrients that are released from dead biomass (Petkovic et al. 2021). Another explanation could be the digestion of storage lipids (Aguilar et al. 2017). After a linear decrease in the cultivation with 300 rpm and 1 vvm, oxygen availability was low (pO_2_ < 5%) after 12 h. For the cultivations with 1 vvm, no oxygen limitation was detected above 600 rpm. Interestingly, in the test series with 2.5 vvm, the same was observed only for the experiments with impeller speeds above 1000 rpm despite the higher gas flow. There was no foam formation observed which could affect mass transfer.

Regardless of the gas flow, both the highest itaconic acid concentration of 26.3 g ∙ L^−1^ (1 vvm) and 22.9 g ∙ L^−1^ (2.5 vvm) and the highest itaconic acid yield of 0.48 g ∙ g^−1^ (1 vvm) and 0.47 g ∙ g^−1^ (2.5 vvm) was achieved at an impeller speed of 300 rpm for both test series. At the same time, the lowest amount of biomass was produced under these conditions, with an CDW of 5.05 and 6.55 g·L^−1^, respectively. Generally, a higher impeller speed led to a higher biomass concentration, reaching an CDW of up to 9.61 g·L^−1^ (1 vvm, 1000 rpm). However, both final product concentration and itaconic acid yield decreased at higher impeller speeds. At the same time, a higher gas flow does not necessarily cause a higher itaconic acid yield or concentration, the comparison of the two test series does not demonstrate a clear correlation between the product formation and the gas flow. The same applies for the biomass production. In contrast to the final concentration, the maximum space time yield after 40 h of 0.24 g ∙ L^‐1^ ∙ h^−1^ (1 vvm) and 0.27 g ∙ L^‐1^ ∙ h^−1^ (2.5 vvm) was not achieved at 300 rpm, but at higher impeller speeds for both gassing rates. Jost et al. observed a similar correlation between oxygen availability and the production of biomass and succinic acid using the obligatory aerobic yeast *Yarrowia lipolytica*. When providing a constant gas flow of 0.4 L∙min^−1^ during the fermentation, DO fell to 0% after 24 h. This led to a 528% increase in product formation and a reduction of 70% of the biomass concentration compared to a fermentation with a constant DO of 50% (Jost et al. 2015). Krull et al. also reported an increased itaconic acid production together with a reduced biomass concentration with lower aeration rates using *Ustilago rabenhorstiana*. Increasing the gas flow from 0.1 to 1.0 vvm increased the biomass concentration by 36%, while reducing the product concentration by 21% (Krull et al. 2020). In this study, increasing the gas flow from 1 vvm to 2.5 vvm at 300 rpm lead to a biomass increase of 14%, and a reduction in product concentration by 13%.

To benchmark the strategy of constant impeller speed and allow for comparison with the common literature approach, a cultivation controlled at a constant DO level of 30% was also performed. The DO setpoint was reached after 13 h, triggering an increase in impeller speed from 300 rpm to a maximum of 929 rpm after 13 h, followed by a gradual decrease to 53 rpm by 140 h. Although oxygen availability was successfully maintained, the resulting itaconic acid concentration and yield were similar to results achieved at higher impeller speeds, but lower than those at a constant impeller speed of 300 rpm. This outcome further supports the conclusion that lower oxygen availability favors itaconic acid production while limiting biomass formation. Given that similar productivities can be achieved at constant impeller speed and under DO control with varying speeds, a strategy employing a constant impeller speed is recommended due to its operational simplicity.

**Figure 2:**
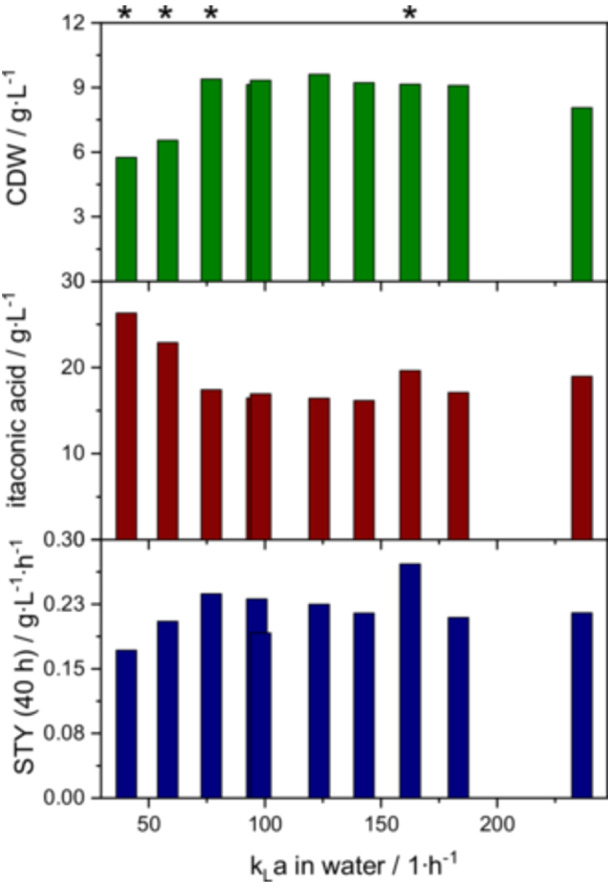
Results of the screening experiments from Table 1 in correlation to the k_L_a value. For every screening experiment, max. CDW, final itaconic acid concentration and STY (40 h) are plotted against the respective k_L_a value of the experiment. Fermentation conditions of screening experiments used for scale up are marked with *. *n* = 1.

The results from lab scale fermentations are highly promising for scale‐up, as high aeration rates and impeller speeds are either costly or just impossible to reach. Additionally, employing a constant impeller speed can simplify process control and reduce operational complexity. A scale up to a 30 L scale using the k_L_a value as scale up criterion was chosen based on the demonstrated influence of oxygen availability on *U. maydis*. Therefore, the screening conditions were characterized with regard to the k_L_a. Interestingly, a correlation can be drawn between the k_L_a value and both the biomass and the product formation. This is shown in Figure 2, where the results (CDW, final concentration, and STY after 40 h) of the screening experiments were plotted against the respective k_L_a value of the experimental conditions.

A clear trend towards a higher biomass concentration with higher k_L_a can be observed until a k_L_a of 77 h^−1^. Despite the aerobic metabolism, the highest itaconic acid concentration was achieved at the relatively low k_L_a value of 40 h^−1^. This hints to a shift in metabolic flux, which is discussed in the last chapter. An increase of the k_L_a, caused by either higher gas flow or higher impeller speed, does not increase the final itaconic acid concentration. However, the space time yield after 40 h does increase further with a higher k_L_a, with an outlying maximum STY (40 h) at 163 h^−1^. But similar to the behavior of the biomass concentration, no clear trend for an increasing STY (40 h) for k_L_a values above 77 h^−1^ could be identified. The most prominent finding when correlating the screening results with the respective k_L_a values is the high itaconic acid concentration at a relatively low k_L_a value.

### 3.2 Scale Up via Different Criteria

The results from the screening experiments lead to a conflict of objectives with regard to the scale up, as the highest itaconic acid yield and the highest STY (40 h) were obtained under different conditions. This is why different operating points were used for the scale up (labeled in Figure 2 with *). Both screening conditions leading to the highest product yield and the highest STY were used, as well as a third point which forms a compromise between the two criteria. As mentioned above, the k_L_a value was selected as scale up criterion. Additionally, the impeller tip speed (ITS) was used as scale up criterion for comparison. Both k_L_a and ITS are commonly used criteria for the scale up of aerobic fermentation processes because they directly influence oxygen transfer efficiency and mixing intensity, which is critical for microbial growth and productivity. Maintaining a similar k_L_a and impeller tip speed helps to ensure consistent oxygen and shear conditions (Bernemann et al. 2024; Margaritis and Zajic 1978). The results of the performed scale up cultivations, together with the screening conditions on which the scale up was based on, are shown in Table 2.

**Table 2:** Summary of the scale up experiments at a gas flow of 1 vvm and impeller speeds from 114 to 469 rpm in 30 L scale and comparison with the respective screening experiment on which the scale up was based.

Impeller speed of scale up experiment	1 vvm, 300 rpm		229 rpm		469 rpm
Conditions of screening experiment on which scale up is based	114 rpm	175 rpm	1 vvm, 600 rpm	2.5 vvm, 300 rpm	2.5 vvm, 900 rpm
**Focus of scale up**	High product yield		Compromise between STY and product yield		high STY
**Scale up criterion**	ITS	k_L_a	ITS	k_L_a	k_L_a
**k**_**L**_ **a/h^−^^1^**	26.0 ± 0.2	39.8 ± 1.0	57.9 ± 1.7		162.5 ± 1.4
**ITS/m·s^−^^1^**	0.63	0.96	1.26		2.58
**Final concentration/g** ∙ **l^−^^1^**	21.36	16.66	16.56		15.37
**Final concentration in screening**	26.3		17.1	22.9	19.6
***Deviation/%***	*18.7*	*36.7*	*3.2*	*27.7*	*21.6*
**max. CDW**	5.05	8.80	8.84		8.65
**max. CDW in screening**	5.75		9.34	9.40	9.16
***Deviation/%***	*12.1*	*52.8*	*3.8*	*6.1*	*5.6*
**Product yield/g**_**ITA**_ **· g**_**GLU**_ **^−^^1^**	0.43	0.33	0.32		0.32
**Product yield in screening**	0.48		0.35	0.47	0.35
***Deviation/%***	*10.4*	*31.3*	*3.0*	*31.9*	*8.6*
**STY (40 h)/g · l^−^^1^ · h^−^^1^**	0.18	0.19	0.21		0.18
**STY (40 h) in screening**	0.17		0.24	0.21	0.27
***Deviation/%***	*5.9*	*11.8*	*12.5*	*0.0*	*33.3*
**Specific product formation rate/g**_**ITA**_ **· g**_**CDW**_ **^−^^1^ · h^−^^1^**	0.069	0.049	0.049		0.046
**product yield per biomass/g**_**ITA**_ **· g**_**CDW**_ **^−^^1^**	7.39	3.10	2.76		2.55

*Note:* The scale up experiments at 114 rpm and 175 rpm were based on the same screening experiment (1 vvm, 300 rpm) but with different scale up criteria. The scale up experiment at 229 rpm was the result of the scale up of 1 vvm and 600 rpm via ITS and of 2.5 vvm and 300 rpm at the same time. For every scale up experiment, the deviation from the underlying screening experiment is given. *n* = 1.

Abbreviations: CDW, cell dry weight; GLU, glucose; ITA, itaconic acid; ITS, impeller tip speed; STY, space time yield.

The screening experiment with the highest product yield (1 vvm, 300 rpm) is scaled up via both impeller tip speed and k_L_a (114 rpm and 175 rpm in 30 L scale). The screening conditions of 1 vvm and 600 rpm resulted in the second highest STY with a moderately high product yield at the same time, which might constitute a promising compromise between the two objectives. A scale up of this operating point via ITS results in an impeller speed of 229 rpm at the 30 L scale. The screening experiment with 2.5 vvm and 300 rpm resulted in the second highest product yield of all tested conditions. Scaling up this operating point via k_L_a results in the same impeller speed of 229 rpm at the 30 L scale. An impeller speed of 469 rpm at the 30 L scale is the scale up of the screening conditions with the highest STY via k_L_a. While a high productivity shortens fermentation time and in consequence leads to lower operational costs, a high final product concentration reduces downstream processing. Therefore, a techno‐economic analysis needs to be performed before industrially relevant fermentations to maximize overall process efficiency.

The deviation in the maximum CDW, the itaconic acid yield and STY of the scale up from the underlying screening experiment lies between 3.0% and 12.1% for ITS as scale up criterion, and between 3.2% and 18.7% for the final itaconic acid concentration. Although some parameters show a lower deviation when applying k_L_a as criterion, the range of deviations here goes up to 52.8%. This shows that the ITS is the preferred scale up method which leads to reproducible results of the screening experiments. Reasons for the low validity of the scale up via k_L_a could be an imprecise correlation of the two reactor systems. Sparger, impeller and oxygen probe are not positioned at exactly the same place. It is assumed that the reactor is not perfectly mixed as this is usually only the case in mathematical models. Therefore, the positioning of the elements is likely to have an influence on the k_L_a value. As the k_L_a is affected by every aspect of the reactor geometry while the ITS is a fixed value without dependencies on the surroundings, the positioning of the reactor internals has no effect on the ITS, but a strong effect on the k_L_a. Additionally, the scale up was based on k_L_a values determined in water. In a previous experiment in the 0.4 L scale it was shown that the k_L_a in water corresponds well with the k_L_a in standard medium (data not shown). However, this correlation is especially close for impeller speeds above 400 rpm in the small scale; the previously performed experiments showed that below this value, the k_L_a in different media deviates stronger. Therefore, it is possible that the oxygen transmission rates were not identical in the two different setups when choosing the k_L_a as scale up criterion. The deviations between the mass transfer coefficients could explain the higher deviations between the experiments at the different scales when scaling up based on the k_L_a value. Calculated according to Doran, impeller flooding occurs in the 30 L scale for impeller speeds below 200 rpm (Doran 2013). This might account for the differing k_L_a values. Generally, the results of the experiments in 30 L scale confirm the findings in the screening experiments where a higher product concentration is achieved with a lower oxygen availability.

**Figure 3:**
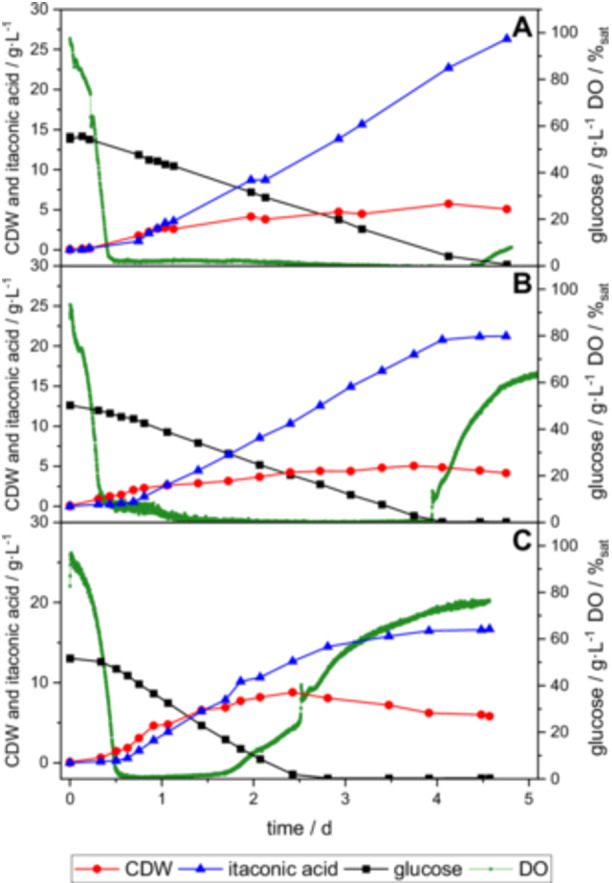
Course of fermentation for screening in 0.4 L at 300 rpm and 1 vvm (A), scale up to 30 L based on impeller tip speed with 114 rpm and 1 vvm (B), as well as scale up based on k_L_a value with 175 rpm and 1 vvm (C). For these fermentations CDW, DO, glucose and itaconic acid concentration are plotted against time.

The scale up using impeller tip speed (see Figure 3B) closely mirrored the pattern observed in the underlying screening experiment conducted at 1 vvm and 300 rpm (Figure 3A). Glucose depletion followed a linear trajectory over 96 h, during which biomass levels steadily increased, reaching an CDW of 5.05 g·L^−1^. Itaconic acid production began after 20 h, rising linearly to a final concentration of 21.4 g ∙ L^−1^. A phase of low oxygen availability commenced at 12 h, persisting to 96 h until glucose was depleted, consistent with the screening fermentation. In contrast, the scale up based on k_L_a showed distinct dynamics (see Figure 3C). Glucose was depleted more rapidly, within 60 h, accompanied by biomass growth to an CDW of 8.80 g·L^−1^ and an itaconic acid production reaching a final concentration of 16.7 g ∙ L^−1^. Unlike the other fermentations, this process exhibited a clear transition in the growth phase after 24 h, shifting from exponential to linear growth as described by Klement et al. (Klement et al. 2012). The low oxygen availability phase in the k_L_a based scale up lasted only from 10 h until 46 h. Despite differences in fermentation profiles and final product concentrations, the STY remained similar across experiments: 0.17 g ∙ L^−1^ ∙ h^−1^ for the screening experiment, 0.18 g ∙ L^−1^ ∙ h^−1^ for the ITS‐based scale up and 0.19 g ∙ L^−1^ ∙ h^−1^ for the k_L_a‐based scale up. Analysis of the product formation rate per biomass shows that the productivity of the biomass under low oxygen conditions is increased compared to the experiment with a higher k_L_a. The product yield per biomass of the experiment with the lowest k_L_a is 2.9 times higher than the product yield per biomass of the experiment with the highest k_L_a. The comparison of the fermentation courses of the two scale up experiments with the underlying screening experiment underscores the suitability of the ITS as scale up criterion to obtain reproducible results. However, this can only be stated for similar impeller configurations as it is the case in these experiments. Simultaneously, the comparison highlights the enhanced itaconic acid production during low oxygen availability. The relatively low k_L_a value of 26 h^−1^ of the experiment with 114 rpm induces to a phase of low oxygen availability, resulting in the highest product concentration among all scale up experiments. The data in Table 2 corroborate these findings, demonstrating that low k_L_a values are associated with higher itaconic acid concentrations, while higher k_L_a values correlate with a shorter phase of low oxygen availability and an increased biomass production.

### 3.3 Influence of Power Input on Productivity

**Figure 4:**
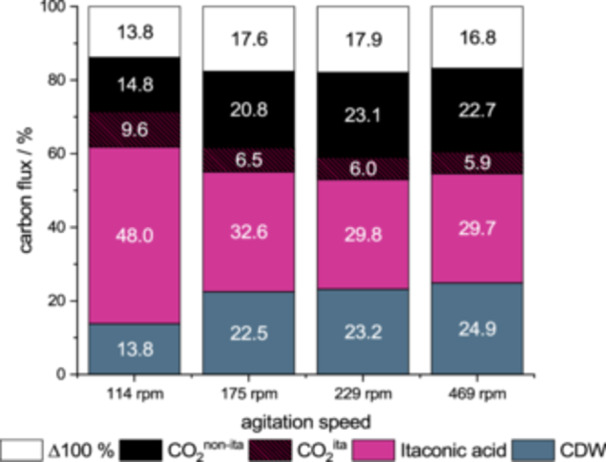
Carbon balance of the fermentations in 30 L scale, broken down by impeller speed. Difference to the total amount of carbon from glucose used (white), carbon in itaconic acid (violet), carbon bound in biomass (gray), CO_2_ from itaconic acid production (violet‐black) and non‐itaconic CO_2_ emission (black).

The trend described in the previous chapter is also clear when analyzing the carbon mass balance at the time of glucose depletion, as shown in Figure 4. The flux of carbon into CDW, itaconic acid and CO_2_ were compared for different agitation speeds in the large‐scale bioreactor. For fermentations with agitation speeds of 175 rpm and higher, the end distribution of carbon was similar. A mean flux of 23.5% carbon to CDW and 30.7% carbon to itaconic acid was observed when applying agitation speeds above 175 rpm For the fermentation performed at 114 rpm, carbon flux is differently distributed. Flux increased towards itaconic acid with 48.0% carbon and decreased towards biomass with only 13.8% carbon. Although, the data set is insufficient for statistical analysis, the observations in the large scale agree with those in the small scale (see Table 1). Together the results show that under low oxygen availability, there is a shift from using the carbon source for biomass accumulation towards itaconic acid production.

The total flux to CO_2_ showed a mean of 27% over all agitation speeds. However, the decarboxylation of trans‐aconitate to itaconic acid, also produces CO_2_. Subtracting “itaconic” CO_2_, the fermentation at 114 rpm had a flux of 14.8% carbon to CO_2_, while the mean flux was 22% at higher agitation speeds. These “non‐itaconic” CO_2_ flux distributions show the same trend as the distribution of biomass flux. In fact, over all agitation speeds the quotient of “non itaconic” CO_2_ to biomass is rather constant with 0.98% ± 0.07%. This indicates a coupling of CO_2_ formation and biomass formation, with a generation of roughly 1 mol of CO_2_ per 1 mol of biomass.

The increased flux towards itaconic acid under oxygen limited availability is not in agreement with common suggestions in the literature, where the importance of oxygen supply for the itaconic acid production of *U. maydis* is often highlighted (Hartmann et al. 2018; Guevarra and Tabuchi 1990; Klement et al. 2012; Tehrani et al. 2019). This is in part attributed to the finding on itaconic acid production with *A. terreus*. In the literature, similar oxygen metabolism is assumed between *A. terreus* and *U. maydis*. Shin et al. reported that *A. terreus* has a critical DO of 15%, below which a reduced production of itaconic acid took place (Shin et al. 2013). However, this has not been investigated in a similar manner for *U. maydis* (Kuenz and Krull 2018). Most experiments in literature are focused on short interruptions of oxygen supply (Hartmann et al. 2018; Guevarra and Tabuchi 1990) or discuss oxygen consumption while changing other parameters as well (Klement et al. 2012; Hartmann et al. 2018). In other cases, the discussion is focused on inhomogeneous distribution of oxygen in the media (Tehrani et al. 2019). In the case presented here, due to the continuous gas flow of 1 vvm and the constant agitation, a long‐term, constantly low oxygen availability was present at lower agitation speeds, see Figure 3. This clearly distinguishes the experiments shown here from the work in which the oxygen supply was switched off for a short time.

In general, introduction of low oxygen conditions to aerobic microorganisms are associated with high costs for the organisms through the necessary metabolic adaptation, for example the energy metabolism (Ernst and Tielker 2009). Therefore, keeping the oxygen availability on a constantly low level might allow an adaptation with comparably high productivity as demonstrated here, while adapting to changing conditions might inhibit productivity. While dissolved oxygen was undetectable at times in the presented fermentations, the ongoing gas flow provided enough oxygen for the organism to avoid complete oxygen depletion. This suggests that the conditions in this study were hypoxic rather than anoxic.

Responses to oxygen limited conditions have been researched in several fungi. As mentioned, comparisons are often made with itaconic acid production in *A. terreus*. However, the critical DO for itaconic acid production is higher than that for biomass formation in *A. terreus* (Shin et al. 2013). This is in contrast to the observations made with *U. maydis* in this study. Therefore, a more general comparison to similar observations might give an insight into the adaptation of *U. maydis* to oxygen limitation.

As anaerobic growth so far has not been described for *U. maydis*, a comparison can be made to obligatory aerobic fungi, such as *Trichoderma reesei*, *Aspergillus nidulans* and *Aspergillus oryzae*. In the work of Bonaccorsi et al. *T. reesei* was subjected to hypoxia. In contrast to anoxia, which describes the total lack of oxygen, in hypoxic conditions oxygen is still present, but in insufficient amounts. Under hypoxia, among others, expression of genes for protein synthesis and cell division were downregulated in *T. reesei* (Bonaccorsi et al. 2006). A similar scenario might be observed here under low oxygen concentrations that do not foster growth. However, this does not explain the increased carbon flux towards itaconic acid.

Formation of itaconic acid leads to a net production of NADH, which needs to be reduced by oxygen. Assuming the theoretical route from glucose, 3 mol NADH are formed per mol of itaconic acid, see Figure 1. In comparison, 10 mol NADH and 2 mol FADH_2_ are formed during cellular respiration of glucose to CO_2_. Cutting the TCA short by exporting cis‐aconitate from the mitochondrion and converting it to itaconic acid, prevents the further production of reduced cofactors. This could be an avoidance strategy of NADH production similar to those observed in filamentous fungi like *A. nidulans* and *A. oryzae*, for example, through the γ‐aminobutyrate (GABA) shunt (Shimizu 2018). Upon entering hypoxic conditions, GABA shunt associated genes were upregulated in these organisms, which lead to a bypass of the NADH forming step from α‐ketoglutarate to succinate in the TCA. This bypass avoids the production of 1 mol NADH, compared to the regular TCA reactions (Hillmann et al. 2015; Masuo et al. 2010; Terabayashi et al. 2012). The increased carbon flux towards itaconic acid under low oxygen availability in *U. maydis* may similarly suggest a use of the itaconic acid pathway as a mechanism to reduce the production of reduced cofactors, and thus the demand for oxygen in cofactor regeneration. To verify this thesis, further research concerning the metabolic processes in *U. maydis* under low oxygen conditions is necessary.

## 4 Conclusion

The present study highlights the complex interplay of parameters impacting the cultivation of *Ustilago maydis* and its scale up for the production of itaconic acid. Lab scale experiments in 0.4 L scale identified optimal conditions for maximizing product concentration, while also addressing the previously unclear influence of DO on itaconic acid production in *U. maydis*. A correlation between k_L_a and biomass growth, final itaconic acid concentration, and space time yield could be established. Notably, a higher k_L_a correlated with increased biomass production and space time yield after 40 h, but it did not enhance final product concentration. To meet the conflict of objectives between high product concentration and high space time yield for the scale up to 30 L scale, scale ups based on different operating points were performed. Using a constant impeller tip speed as scale up criterion provided greater consistency in replicating the screening results compared to a k_L_a ‐based scaling, which exhibited wider variability, possibly due to different positioning of the oxygen probe. These findings recommend the use of impeller tip speed as a more reliable criterion, enabling scalable and reproducible production of itaconic acid independent of the reactor geometry. The finding from the screening experiments that a lower k_L_a value correlates with an increased final product concentration was confirmed in the scale up experiments. This was also underlined by a carbon balance analysis. In experiments with a lower impeller speed, which is associated with a lower k_L_a, a higher carbon share based on the substrate glucose was directed into the product itaconic acid, while a higher impeller speed shifted the flux to a higher biomass concentration. The enhanced production of itaconic acid could be a mechanism to bypass NADH producing steps of the TCA under conditions with low oxygen availability. The results presented in this study stand in clear contradiction to previously published literature. Due to the consistent biological replicates, the results are nevertheless considered reliable. Further research is needed to understand the metabolic processes in *U. maydis* under these conditions. Particularly, analyses of metabolic fluxes, cofactor balances, and gene regulation under limited oxygen availability are necessary to fully understand the underlying mechanisms. Another aspect that needs to be analysed to further explore the metabolic processes in *U. maydis* is the ratio between carbon and nitrogen sources under low oxygen conditions. Usually, a high carbon to nitrogen ratio is required for itaconic acid production. Especially when using complex media derived from renewable resources with only limited influence on the exact composition, the oxygen regime could be a valuable instrument to manipulate the metabolism of *U. maydis* towards increased itaconic acid production. Oxygen availability might be an additional tool to influence the balance between the production of biomass and itaconic acid in favor of product formation for an increased product yield.

## Author Contributions

**Marianne Volkmar:** conceptualization, data curation, formal analysis, investigation, methodology, visualization, writing – original draft preparation, writing – review and editing. **Wolfgang Laudensack:** conceptualization, data curation, formal analysis, investigation, methodology, visualization, writing – original draft preparation, writing – review and editing. **Felix Bartzack:** data curation, formal analysis, investigation, methodology, visualization, writing – original draft preparation. **Niklas Erdmann:** conceptualization, data curation, formal analysis, writing – original draft preparation, writing – review and editing. **Sonja Schönrock:** conceptualization, writing – original draft preparation, writing – review and editing. **Emely Fuderer:** conceptualization, writing – original draft preparation, writing – review and editing. **Dirk Holtmann:** conceptualization, funding acquisition, project administration, supervision, writing – review and editing. **Lars M. Blank:** conceptualization, resources, supervision, writing – review and editing. **Roland Ulber:** conceptualization, funding acquisition, project administration, resources, supervision, writing – review and editing.

## Conflicts of Interest

The authors declare no conflicts of interest.

## Supporting information


**Figure S1:** Sketch of the Biostat Q+ (A) and the Biostat C+ (B) test setups with the geometrical dimensions of the reactor internals.

## Data Availability

The data that support the findings of this study are available from the corresponding author upon reasonable request.
