# Supplementary material for: Low Oxygen Availability Increases Itaconate Production by Ustilago maydis
Source: Biotechnol Bioeng. 2025 Aug 2;122(11):3007–17. doi: 10.1002/bit.70035 (PMC12503012; doi:10.1002/bit.70035)
Supplement: Supplementary file 1 — Figure S1: Sketch of the Biostat Q+ (A) and the Biostat C+ (B) test setups with the geometrical dimensions of the reactor internals. [file BIT-122-3007-s001.docx]

# Supplementary material


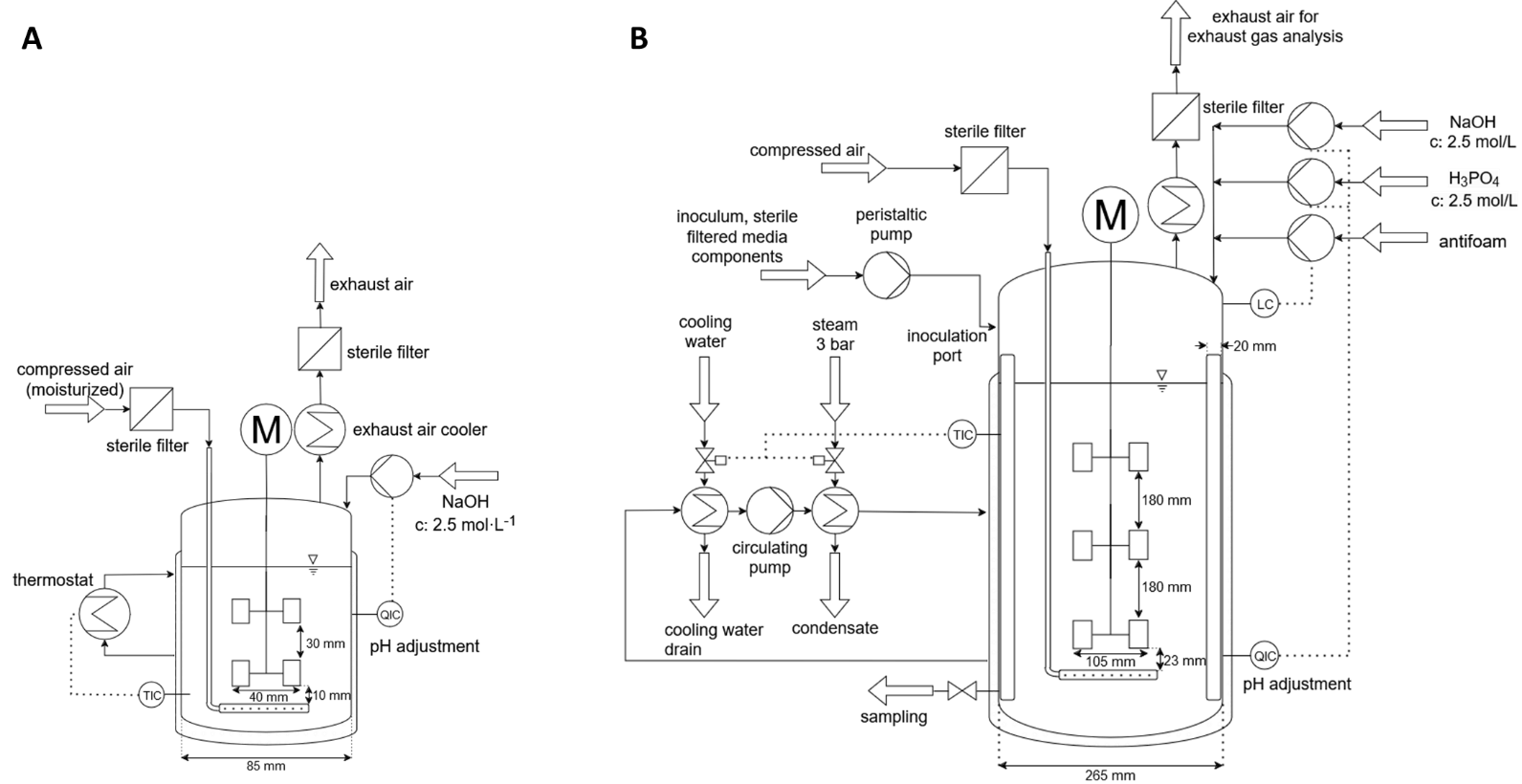


Figure S1: Sketch of the Biostat Q+ (A) and the Biostat C+ (B) test setups with the geometrical dimensions of the reactor internals.
